# Recent advances and applications of nitroreductase activable agents for tumor theranostic

**DOI:** 10.3389/fphar.2024.1451517

**Published:** 2024-07-19

**Authors:** Baoxin Shang, Zongjiang Yu, Zhengdan Wang

**Affiliations:** ^1^ The Afffliated Hospital of Qingdao University, Qingdao University, Qingdao, China; ^2^ CAS Key Laboratory of Biobased Materials, Qingdao Institute of Bioenergy and Bioprocess Technology, Chinese Academy of Sciences, Qingdao, China; ^3^ Shandong Energy Institute, Qingdao, China; ^4^ Qingdao New Energy Shandong Laboratory, Qingdao, China

**Keywords:** nitroreductase activable agents, tumor theranostics, targeted drug delivery, real-time imaging, enzyme-responsive prodrugs

## Abstract

Nitroreductase activable agents offer a personalized and targeted approach to cancer theranostics by selectively activating prodrugs within the tumor microenvironment. These agents enable non-invasive tumor imaging, image-guided drug delivery, and real-time treatment monitoring. By leveraging the enzymatic action of tumor-specific nitroreductase enzymes, cytotoxic drugs are delivered directly to cancer cells while minimizing systemic toxicity. This review highlights the key features, mechanisms of action, diagnostic applications, therapeutic potentials, and future directions of nitroreductase activable agents for tumor theranostics. Integration with imaging modalities, advanced drug delivery systems, immunotherapy combinations, and theranostic biomarkers shows promise for optimizing treatment outcomes and improving patient survival in oncology. Continued research and innovation in this field are crucial for advancing novel theranostic strategies and enhancing patient care. Nitroreductase activable agents represent a promising avenue for personalized cancer therapy and have the potential to transform cancer diagnosis and treatment approaches.

## 1 Introduction

Tumor theranostics, which combines therapeutic and diagnostic functions in a single platform, represents a promising strategy for improving cancer treatment outcomes (Hapuarachchige and Artemov, 2020; Jiang et al., 2021a; Hu X. et al., 2022; Wu D. et al., 2022; Wang et al., 2022; Zhao et al., 2022; Zhang et al., 2023a; Bose et al., 2023; Qi et al., 2024). Nitroreductase activable agents have emerged as a novel approach in tumor theranostics, harnessing the enzymatic activity of nitroreductases to selectively target and treat cancer cells while enabling real-time imaging of treatment response (Li et al., 2024a; Morsby et al., 2024). NTRs are overexpressed in various cancer types, including colorectal, breast, and liver cancers. The levels of NTR expression in these tumors are significantly higher than the basal levels found in healthy cells, often by several orders of magnitude. This differential expression underpins the selective activation of NTR-targeted therapies, enhancing therapeutic efficacy while minimizing off-target effects (Williams et al., 2015; Hu X. et al., 2022; Qi et al., 2024).

Nitroreductases are enzymes capable of reducing nitroaromatic compounds, leading to the generation of cytotoxic intermediates such as nitroso and hydroxylamine species (Qi et al., 2020). Nitroreductases are commonly found in bacteria, fungi, and some mammalian cells, which have the ability to reduce nitroaromatic compounds to generate reactive intermediates that are toxic to cells (Qiao et al., 2021; Wu Y. et al., 2022; Geng et al., 2024; Xiang et al., 2024). Importantly, the expression of nitroreductases is often elevated in tumor cells compared to normal tissues, making them attractive targets for cancer therapy (Jiang et al., 2023a; Xin et al., 2024). By exploiting the differential expression of nitroreductases, researchers have developed prodrugs that are activated specifically in tumor cells, allowing for precise delivery of therapeutic agents and imaging probes (Searle et al., 2004; Williams et al., 2015; Sharrock et al., 2021).

Nitroreductase activable agents are a class of prodrugs designed to selectively target and treat cancer cells by exploiting the enzymatic activity of nitroreductases. The design of nitroreductase activable agents involves coupling a therapeutic or imaging agent to a nitroaromatic group that can be selectively activated by nitroreductase enzymes (Li et al., 2022; Skorjanc et al., 2023; Wang et al., 2023; Li et al., 2024b; Geng et al., 2024). Upon enzymatic reduction of the nitro group, the prodrug is cleaved to release the active therapeutic or imaging moiety specifically within tumor cells, leading to localized treatment effects. This targeted activation enhances the therapeutic index of the agents, as they are designed to exert their effects predominantly in cancerous tissues while sparing normal cells from toxicity.

In conclusion, nitroreductase activable agents represent a promising strategy in tumor theranostics, offering a multifaceted approach to cancer treatment that combines targeted therapy with real-time imaging capabilities. The development of these agents holds great potential for advancing personalized cancer care and improving clinical outcomes for patients. This comprehensive review aims to elucidate the current state of research in this field and highlight the future directions and opportunities for utilizing nitroreductase activable agents in cancer theranostics.

## 2 Distinct advantages of nitroreductase activable agents

The concept of nitroreductase activable agents for tumor theranostic purposes encompasses two main components: therapeutic agents and diagnostic probes. Therapeutic agents, such as chemotherapeutic drugs or cytotoxic compounds, are conjugated to a nitroreductase-sensitive moiety that is cleaved upon enzymatic activation within tumor cells (Jiang et al., 2021b; Jiang et al., 2023a; Jiang et al., 2023b; Zhang et al., 2023b; Zhao et al., 2023). This targeted release of active therapeutics enhances the efficacy of cancer treatment while minimizing systemic toxicity (Knox et al., 1995; Güngör et al., 2019; Güngör et al., 2020; Tokay et al., 2020). In parallel, diagnostic probes, including fluorescent dyes, imaging agents, or nanoparticles, can be incorporated into the prodrug structure to enable real-time monitoring of drug activation and tumor response to therapy (McNerney et al., 2021; Rajapaksha et al., 2024; Yin et al., 2024).

The therapeutic potential of nitroreductase activable agents lies in their ability to selectively target tumor cells, bypassing the inherent heterogeneity and drug resistance commonly observed in cancer. By exploiting the tumor-specific expression of nitroreductases, these agents offer a precision medicine approach to cancer treatment, tailoring therapy to individual patient characteristics. Additionally, the integration of diagnostic components allows for the non-invasive monitoring of treatment response, enabling clinicians to adapt therapeutic strategies and optimize patient outcomes.

Overall, nitroreductase activable agents represent a versatile and promising approach in tumor theranostics, offering a combination of targeted therapy and diagnostic capabilities for cancer treatment. The specific activation of these agents within tumor cells allows for precise drug delivery and imaging, enhancing the therapeutic outcomes and patient care in oncology. Continued research and development in this field hold great potential for advancing personalized cancer therapy and improving clinical outcomes for cancer patients.

## 3 Examples of nitroreductase activable agents for tumor theranostic

Nitroreductase activable agents for tumor theranostics can be broadly categorized into two main types: small molecule theranostic agents and integrated delivery systems.

### 3.1 Single small molecule theranostic agents

This approach involves the fusion of therapeutic and diagnostic moieties within a single small molecule or nanoplatform. These agents are designed to be selectively activated by nitroreductase enzymes within the tumor microenvironment, thereby releasing their therapeutic and diagnostic functions specifically at the tumor site.

**FIGURE 1:**
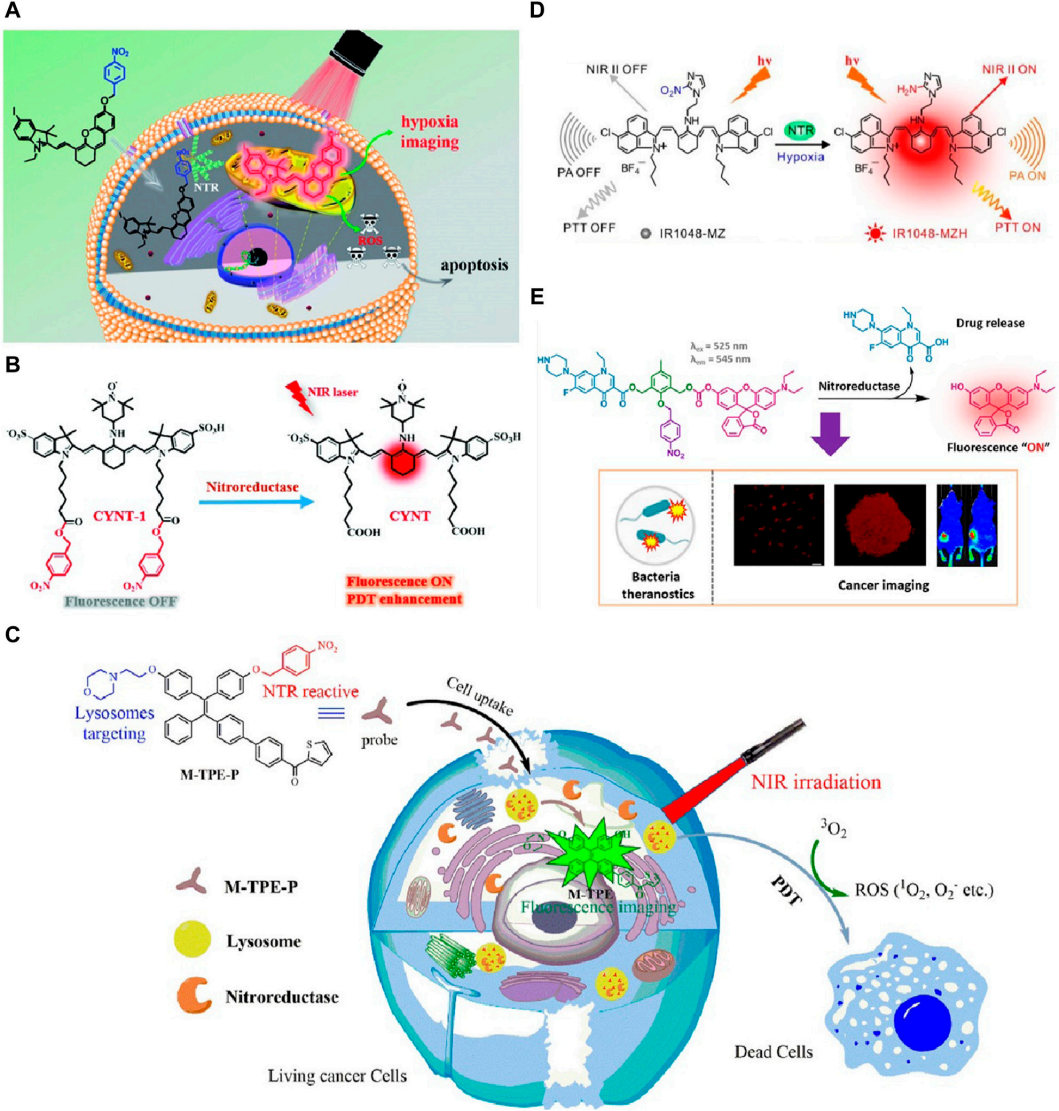
**(A)** ICy-N strategy designed for hypoxia detection and cancer treatment. Adapted with modification from Ref (Xu et al., 2019). ^©^ 2019 Wiley-VCH GmbH. **(B)** Structures of CYNT-1 and CYNT, including their mechanisms for sensing and PDT activation. Adapted with modification from Ref (Zheng et al., 2020). ^©^ 2020 Wiley-VCH GmbH. **(C)** Diagram of the NTR-responsive photosensitizer that turns on fluorescence for imaging lysosomes and performing PDT. Adapted with modification from Ref (Wang et al., 2024). ^©^ 2024 Elsevier. **(D)** Activation mechanism of IR1048-MZ by NTR, accompanied by the frontier molecular orbitals of IR1048-MZ and IR1048-MZH (Meng et al., 2018). **(E)** Enzyme-catalyzed activation process of NR-NO2 mediated by NTR. Adapted with modification from Ref (Karan et al., 2023). ^©^ 2023 American Chemical Society.

Peng’s group in 2019 developed a hypoxia-activated near-infrared (NIR) photosensitizer, ICy-N, for *in vivo* cancer treatment (Xu et al., 2019). ICy-N was non-fluorescent and low in singlet oxygen production in normal tissues but was activated by nitroreductase in tumor regions, producing strong fluorescence and singlet oxygen for photodynamic therapy (PDT). The reduced product, ICy-OH, localized in mitochondria, efficiently induced cell apoptosis under 660 nm light (Figure 1A). *In vivo* studies in Balb/c mice demonstrated precise tumor hypoxia imaging and effective tumor growth inhibition, highlighting the critical role of nitroreductase in theranostics. Furthermore, such PDT therapy coupled with fluorescent imaging tactic for tumor theranostic has also been witnessed in several other studies. However, the delivery efficiency of ICy-N could be improved, as some tumors may exhibit varying levels of hypoxia, affecting the activation and therapeutic outcome. Zheng et al. (2020) developed a nitroreductase-activatable near-infrared theranostic photosensitizer, CYNT-1, for PDT. Nitroreductase activated CYNT-1 in mildly hypoxic tumor regions, causing fluorescence and singlet oxygen production, enabling precise imaging and efficient PDT (Figure 1B). This enhanced treatment specificity and minimized side effects, showing great clinical potential. Notably, CYNT-1 faces challenges in maintaining a high signal-to-noise ratio of imaging signals under varying tumor microenvironments. Li et al. (2021) developed a nitroreductase-responsive NIR phototheranostic probe, HCN, for *in vivo* imaging and PDT of tiny tumors. Nitroreductase catalyzed the reduction of the probe’s nitro group to an amino group, activating its fluorescence and photosensitizing properties. This enabled precise imaging of tumors as small as 6 mm³ and effective PDT under hypoxic conditions. Furthermore, the stability of the NIR phototheranostic probe HCN under physiological conditions needs further optimization for prolonged circulation *in vivo*. The probe demonstrated high sensitivity and selectivity to nitroreductase, allowing for the dynamic monitoring of tumor hypoxia and significantly improving treatment specificity and minimizing side effects. In Liu and colleges’ design in 2019, the nitroreductase activated the theranostic molecule under mild hypoxia via a photoinduced electron transfer mechanism, enhancing fluorescence and photodynamic therapy (PDT) efficiency (Liu et al., 2019). This process involved reducing nitro groups to amines, activating singlet oxygen production, which disrupted lysosomes and improved tumor imaging and treatment. It is worth mentioning that, the photoinduced electron transfer mechanism of the theranostic molecule may result in inconsistent therapeutic outcomes under different hypoxic conditions. Digby and coworkers in 2022 synthesized a NTR activated theranostic agent, which could reduce the nitro group to an amine, triggering chemiluminescence resonance energy transfer (CRET) and the production of singlet oxygen of the prodrug (Digby et al., 2022). Notably, in this study, NTR enabled a light-independent activation of the photosensitizer, overcoming the limitations of light penetration in traditional PDT. However, the CRET mechanism could suffer from reduced efficiency in deep tissue imaging due to limited light penetration. In Wang’s study in 2024, NTR was used as a biomarker to assess tumor hypoxia (Wang et al., 2024). The designed probe, M-TPE-P, targeted lysosomes, showing high selectivity and sensitivity towards NTR (Figure 1C). Upon NTR detection, the probe became fluorescent, enabling the imaging of lysosomal NTR activity. Additionally, M-TPE-P effectively generated ROS under NIR light, enhancing its potential for PDT in cancer treatment. Notably, the M-TPE-P probe’s selectivity towards lysosomes requires further validation in diverse cancer models to confirm its broad applicability.

Meng and coworkers in 2018 developed a hypoxia-triggered and nitroreductase-responsive single molecule probe, IR1048-MZ, for high-contrast near-infrared (NIR II) and photoacoustic (PA) tumor imaging and photothermal therapy (PTT) (Meng et al., 2018). The probe was activated by NTR in hypoxic tumors, emitting strong NIR II fluorescence and PA signals, enabling precise tumor imaging and deep tissue penetration (Figure 1D). Additionally, IR1048-MZ exhibited significant temperature increases during PTT, effectively ablating tumors without recurrence. This research highlighted the critical role of NTR in theranostics and its promising clinical applications. However, the hypoxia-triggered probe IR1048-MZ may exhibit variable activation efficiency in tumors with heterogeneous hypoxic regions, potentially affecting imaging and therapy accuracy.

In Karan and coworkers’ study in 2023, the NTR-activable prodrug NR-NO2 was developed for the theranostic imaging and treatment of cancer and bacterial infections (Karan et al., 2023). The prodrug comprised an antibiotic (norfloxacin), a chemotherapeutic agent, and a fluorescent dye (Figure 1E). Upon activation by NTR, which is overexpressed in hypoxic tumor environments, NR-NO_2_ exhibited a significant fluorescence “turn-on” effect, allowing real-time monitoring of drug release. It selectively accumulated in tumors, providing enhanced antibacterial and anticancer activity. This innovation demonstrated a promising approach for targeted cancer therapy, leveraging NTR’s role in activating the prodrug under hypoxic conditions, thus enhancing therapeutic efficacy while enabling precise imaging. Furthermore, this prodrug’s fluorescence “turn-on” effect needs enhancement to improve sensitivity and reduce false positives in complex tumor environments. In Chan’s design in 2023, NTR was utilized to activate fluoroquinolone prodrugs, masking the β-ketoacid group to reduce off-target effects (Chan et al., 2023). The NTR-responsive prodrugs selectively released the parent drug in hypoxic tumor environments, minimizing systemic toxicity. This strategy enhanced the antibacterial and anticancer efficacy of fluoroquinolones while preventing Achilles tendon rupture caused by magnesium ion chelation. But the hydrolysis-resistant fluoroquinolone prodrugs require further investigation to minimize potential off-target effects in non-hypoxic tissues. Generally, the innovative design of hydrolysis-resistant prodrugs showed promising therapeutic outcomes by enabling targeted activation in hypoxic conditions, offering a potential advancement in cancer treatment and reducing adverse effects.

### 3.2 Integrated delivery systems

The second approach focuses on developing complex delivery systems that incorporate both therapeutic and diagnostic agents into a single platform but as separate entities. These systems can be designed as nanoparticles, micelles, or other nanocarriers that encapsulate the active agents separately but within the same delivery vehicle.

**FIGURE 2:**
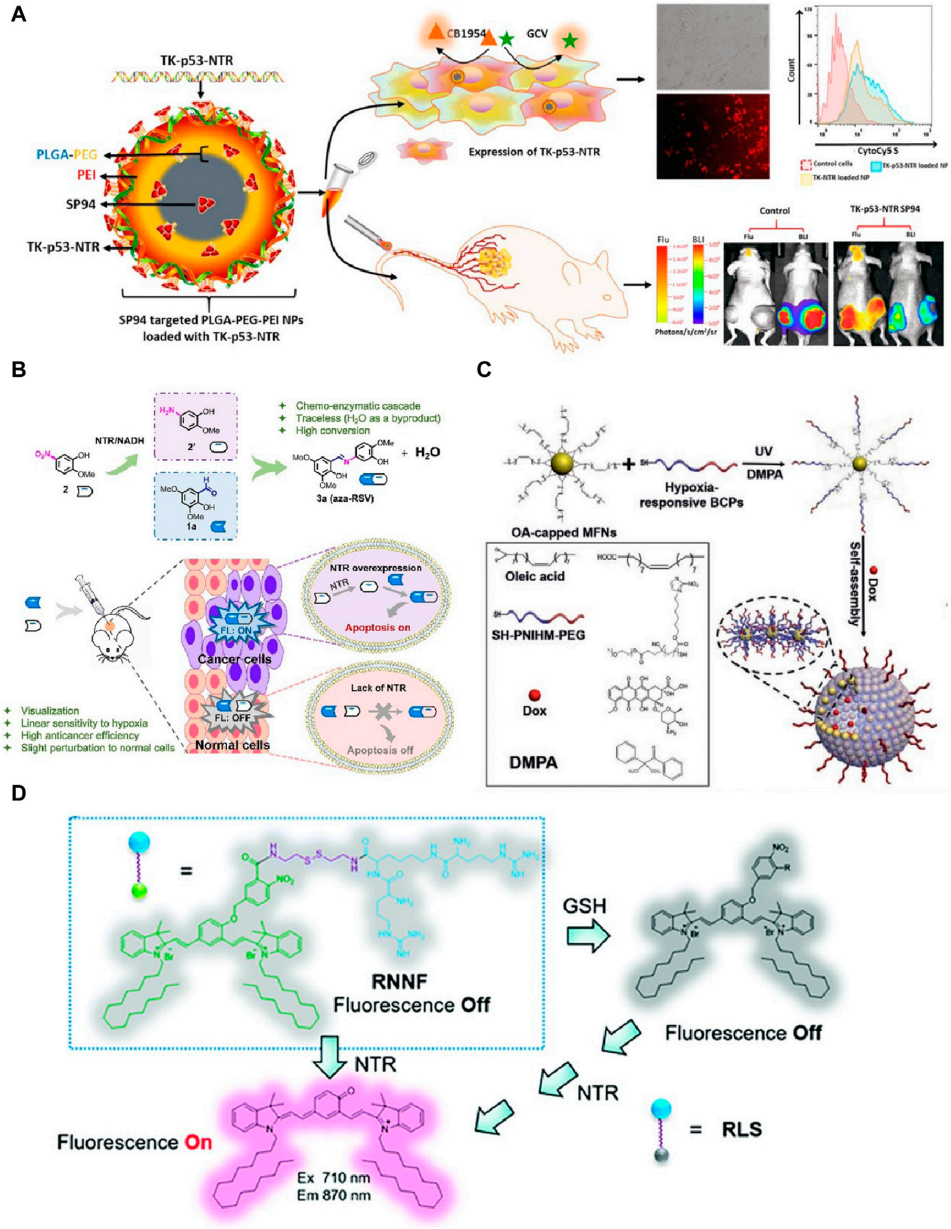
**(A)** Diagram showing the design of TK−p53−NTR triple-gene-loaded PLGA−PEG−PEI nanoparticles functionalized with SP94, their targeted delivery to HCC cells, the sequence of events upon cellular uptake, and their use in bioluminescence imaging of nude mice with HepG2 tumor xenografts. Adapted with modification from Ref (Kumar et al., 2023). ^©^ 2020 American Chemical Society. **(B)** Schematic overview of the activation of the aza-RSV prodrug via NTR-induced bioorthogonal ligation, detailing: **(A)** the formation of aza-RSV 3a through the condensation of aldehyde 1a and amine 2′, derived from nitro substrate 2 in the presence of NTR and NADH; **(B)** depiction of cancer-specific imaging and apoptosis using this prodrug approach. Adapted with modification from Ref (Hu L. et al., 2022). ^©^ 2022Elsevier. **(C)** Illustration of a nanoplatform responsive to both NTR and GSH for gene delivery and fluorescence imaging. Adapted with modification from Ref (Liang et al., 2020). ^©^ 2020 Royal Society of Chemistry. **(D)** Fabrication process of Dox-MVs through the cooperative assembly of BCP-grafted MFNs, and their application for hypoxia-triggered release of Dox and O2 within tumors. Adapted with modification from Ref (Yang et al., 2021). ^©^ 2021 Wiley-VCH GmbH.

Sukumar and colleges in 2020 developed a nanoplatform for tumor theranostic. NTR played a critical role in the theranostic approach for treating hepatocellular carcinoma (HCC) by activating the prodrug CB1954 to produce cytotoxic metabolites (Kumar et al., 2023). The engineered SP94-targeted triblock copolymer nanoparticles efficiently delivered a triple therapeutic gene (thymidine kinase (TK), p53, and NTR) to tumor cells (Figure 2A). This approach restored p53 function, enhanced cancer cell apoptosis, and improved the therapeutic efficacy against both wild-type and mutant p53 HCC cells. Imaging was crucial in evaluating therapeutic efficacy and gene delivery. NTR activated the fluorescent substrate CytoCy5S, emitting red fluorescence upon activation, enabling real-time tracking and visualization of NTR expression within tumor cells. This imaging capability allowed for precise localization and quantification of therapeutic gene delivery *in vivo*, facilitating accurate monitoring of treatment distribution, gene expression levels, and therapeutic outcomes. The innovative combination of targeted delivery and triple gene therapy demonstrated significant tumor reduction, highlighting its potential for effective and targeted cancer treatment. However, this SP94-targeted triblock copolymer nanoparticles face challenges in achieving consistent gene delivery efficiency across different tumor types.

In Hu’s study in 2022, NTR enabled a traceless bioorthogonal ligation strategy to activate a prodrug for cancer-specific imaging and therapy (Hu L. et al., 2022). The prodrug system involved the reduction of nitroaromatic substrates to arylamines by NTR in hypoxic tumor environments, followed by bioorthogonal imination with aldehydes to produce the anticancer agent aza-RSV (Figure 2B). This process generated a specific “turn-on” fluorescence signal, facilitating precise imaging of cancer cells. The innovative approach demonstrated significant anticancer activity under hypoxia with minimal impact on normal cells, highlighting its potential for targeted cancer therapy by providing effective imaging and therapeutic outcomes with high specificity. Notably, the bioorthogonal ligation strategy for activating the prodrug may encounter stability issues under physiological conditions, affecting its therapeutic performance. Alternatively, a nanoplatform responsive to NTR and glutathione (GSH) was developed for gene delivery and near-infrared fluorescence imaging in tumors in 2021 (Liang et al., 2020). The platform integrated indocyanine green analogs and an arginine-rich dendritic peptide, forming assemblies that responded to NTR and GSH by disassembling and releasing their cargo (Figure 2C). NTR triggered a specific fluorescence “turn-on” effect, facilitating real-time imaging of gene delivery. However, the responsiveness of the nanoplatform to both NTR and GSH may lead to premature cargo release, reducing the overall therapeutic efficacy. This system demonstrated high gene transfection efficiency and low cytotoxicity, outperforming commercial reagents. *In vivo*, the platform enabled precise gene expression localization and efficient tumor imaging, showcasing its potential for targeted cancer theranostics.

In another study, a NTR-responsive polymeric micelle system was developed for targeted intracellular release of doxorubicin (DOX) in cancer therapy in 2021 (Sun et al., 2021). The amphiphilic polymer TNP, containing a 4-nitrobenzyl group and aggregation-induced emission (AIE) moieties, self-assembled into micelles encapsulating DOX. NTR, overexpressed in tumor cells, catalyzed the reduction of the 4-nitrobenzyl group, triggering micelle decomposition and DOX release. This process also activated the AIE fluorescence, enabling real-time imaging of drug release. However, the NTR-responsive polymeric micelles may have limited stability in the bloodstream, affecting the controlled release of doxorubicin. The TNP@DOX micelles exhibited high selectivity for cancer cells, minimal toxicity to normal cells, and efficient gene transfection, demonstrating significant potential for precise cancer theranostics with controlled drug release and imaging capabilities. Yang et al. have developed hypoxia-responsive nanovesicles using manganese ferrite nanoparticles (MFNs) for enhanced cancer treatment (Yang et al., 2021). These nanovesicles, loaded with doxorubicin, dissociated under tumor hypoxia, releasing the drug and alleviating hypoxia by decomposing endogenous H_2_O_2_ (Figure 2D). This improved the efficacy of αPD-L1-mediated checkpoint blockade therapy, significantly suppressing tumor growth and preventing recurrence. The innovation lay in combining drug delivery with hypoxia alleviation, enhancing chemotherapy and immunotherapy effectiveness for long-term tumor control. It is worth mentioning that, the hypoxia-responsive nanovesicles need further optimization to improve their dissociation efficiency under tumor hypoxia and enhance their therapeutic outcomes.

In conclusion, nitroreductase activable agents for tumor theranostics represent a highly promising and innovative approach in cancer diagnosis and treatment. These agents leverage the enzymatic activity of nitroreductases to achieve targeted therapy and diagnostic imaging within the tumor microenvironment, providing a non-invasive and precise method for monitoring and treating tumors. Besides the summarized examples above, dual-locked (NTR and another cancer biomarker) activatable theranostics have also been reported and widely used, offering new diagnostic methods (Luo et al., 2022; Wei et al., 2022). By selectively activating prodrugs in tumor cells, these agents enhance therapeutic efficacy while minimizing systemic toxicity. The integration of diagnostic and therapeutic functions enables real-time imaging and image-guided treatment, optimizing patient outcomes. Continued research and clinical development in this field hold significant potential for advancing personalized cancer therapy and improving survival rates.

## 4 Future directions in the development of nitroreductase activable agents for tumor theranostics

The field of nitroreductase activable agents for tumor theranostics is rapidly evolving, with ongoing research focusing on advancing the design, development, and clinical translation of these innovative agents. Several exciting future directions are emerging in this area, which hold great promise for improving cancer therapy and patient outcomes. Here are some key aspects of future directions in the development of nitroreductase activable agents for tumor theranostics.

### 4.1 Enhanced drug delivery systems

Future research efforts will focus on developing advanced drug delivery systems that can improve the tumor-specific accumulation and activation of nitroreductase activable agents. Strategies such as nanoparticle-based drug delivery, targeted drug conjugates, and stimuli-responsive drug release systems will be explored to enhance drug delivery efficiency, increase intratumoral drug concentrations, and minimize off-target effects. By optimizing drug formulation and delivery mechanisms, researchers aim to enhance the therapeutic efficacy and safety of nitroreductase activable agents in cancer treatment.

### 4.2 Multifunctional prodrugs

The development of multifunctional prodrugs that combine multiple therapeutic and diagnostic components within a single agent represents a promising future direction in nitroreductase activable agents. By incorporating imaging agents, targeting ligands, and therapeutic payloads in a single molecular entity, researchers can achieve synergistic effects, improved tumor targeting, and enhanced theranostic capabilities. Multifunctional prodrugs offer a versatile platform for personalized cancer therapy, enabling tailored treatment strategies based on individual patient characteristics and tumor biology.

### 4.3 Immunotherapy combinations

Future studies will explore the integration of nitroreductase activable agents with immunotherapy approaches, such as immune checkpoint inhibitors and chimeric antigen receptor (CAR) T cell therapy, to enhance the anti-tumor immune response. By combining the immunomodulatory effects of immunotherapies with the targeted cytotoxicity of nitroreductase activable agents, researchers aim to boost the immune-mediated tumor killing, overcome treatment resistance, and improve overall treatment outcomes in patients with cancer. Immunotherapy combinations hold great promise for achieving durable responses and long-term survival benefits in cancer therapy.

### 4.4 Theranostic biomarkers

Future research will focus on identifying and validating theranostic biomarkers that can predict treatment response, monitor disease progression, and guide treatment decisions in patients receiving nitroreductase activable agents. Biomarkers such as tumor-specific nitroreductase expression, drug activation levels, and imaging signals can serve as predictive indicators of treatment efficacy and patient outcomes. By incorporating theranostic biomarkers into clinical practice, clinicians can personalize treatment regimens, optimize therapy outcomes, and improve patient care in oncology.

### 4.5 Clinical translation and regulatory approval

An important future direction in the field of nitroreductase activable agents is the translation of preclinical research findings into clinical applications and regulatory approval for clinical use. Clinical trials will be conducted to evaluate the safety, efficacy, and pharmacokinetics of nitroreductase activable agents in cancer patients, with the goal of obtaining regulatory approval for their use in clinical practice. By advancing the clinical translation of these innovative agents, researchers aim to bring novel theranostic approaches to the bedside and improve patient access to advanced cancer therapies.

Generally, the future directions in the development of nitroreductase activable agents for tumor theranostics hold great promise for advancing cancer therapy, personalized medicine, and patient care. Continued research and innovation in this field will drive the development of novel theranostic strategies, optimize treatment outcomes, and ultimately improve the quality of life and survival rates for cancer patients.

## 5 Conclusion

The use of nitroreductase activable agents for tumor theranostics represents a promising and innovative approach in the field of cancer diagnosis and treatment. These agents offer a unique platform for combining diagnostic imaging with targeted therapy, enabling non-invasive tumor monitoring, image-guided treatment, and personalized medicine in oncology. Through the selective activation of prodrugs by tumor-specific nitroreductase enzymes, these agents provide a powerful tool for delivering cytotoxic drugs specifically to cancer cells while sparing normal tissues, thus minimizing toxicity and maximizing therapeutic efficacy.

In this review, we have highlighted the key features, mechanisms of action, diagnostic applications, therapeutic potentials, and future directions of nitroreductase activable agents for tumor theranostics. The ability of these agents to selectively activate prodrugs within the tumor microenvironment offers several advantages, including enhanced tumor targeting, reduced systemic toxicity, and improved treatment responses. By utilizing imaging modalities such as PET, MRI, and fluorescence imaging, clinicians can non-invasively visualize drug activation, monitor treatment responses, assess tumor heterogeneity, and guide treatment decisions in real-time.

Nitroreductase activable agents have shown great promise in preclinical studies and early-phase clinical trials as effective theranostic tools for various types of cancer, including solid tumors and hematological malignancies. The integration of these agents with conventional chemotherapy, radiotherapy, and immunotherapy approaches has the potential to enhance treatment outcomes, overcome treatment resistance, and improve patient survival rates. By developing advanced drug delivery systems, multifunctional prodrugs, immunotherapy combinations, and theranostic biomarkers, researchers aim to further optimize the therapeutic efficacy and clinical utility of nitroreductase activable agents in cancer therapy.

In conclusion, nitroreductase activable agents hold great potential for revolutionizing cancer theranostics by providing a personalized, targeted, and image-guided approach to cancer diagnosis and treatment. Continued research, innovation, and clinical translation in this field are essential for advancing the development of novel theranostic strategies, enhancing treatment outcomes, and improving patient outcomes in oncology. By leveraging the unique properties of nitroreductase activable agents, clinicians can tailor treatment regimens to individual patient needs, optimize therapy responses, and ultimately improve the quality of life and survival rates for cancer patients.
